# Research and practice of flipped classroom based on mobile applications in local universities from the perspective of self-determination theory

**DOI:** 10.3389/fpsyg.2022.963226

**Published:** 2023-01-09

**Authors:** Yan'e Hao, Yongqiang Lan

**Affiliations:** ^1^College of Architecture and Engineering, Yan'an University, Yan'an, China; ^2^Department of Basic Construction, Yan'an University, Yan'an, China

**Keywords:** self-determination theory, mobile application, flipped classroom, basic psychological needs, classroom satisfaction

## Abstract

Self-determination theory is a psychological theory proposed by American psychologists and is widely used in research in the field of education. Mobile applications are gradually changing the traditional classroom communication mode between teachers and students with their intelligent, portable, and humanized operation. Deep integration of information technology and education teaching, promoting mobile applications into the teaching process, facilitating local colleges and universities to better achieve the cultivation goal of high-quality application-oriented talents, and exploring a new learner-centered classroom teaching model are hot issues of current research in the education field. The flipped classroom and mobile application were effectively combined, and a flipped classroom model based on mobile application was proposed and implemented. Based on self-determination theory, this study investigates the current situation of students' basic psychological needs satisfaction and classroom satisfaction under the flipped classroom model based on mobile applications and explores the relationship between the students' basic psychological needs satisfaction and classroom satisfaction. A total of 151 local college students in different professional fields participated in the questionnaire survey. The research results reveal that in the flipped classroom model based on mobile applications, the students' basic psychological needs satisfaction and classroom satisfaction are at a high level, and the two are significantly positively correlated. Therefore, the students' basic psychological needs affect their satisfaction with the classroom, which provides some references for the smooth implementation and further promotion and application of the classroom teaching model.

## 1. Introduction

### 1.1. Self-determination theory

Self-determination theory (SDT) is a comprehensive theory of human motivation proposed by American psychologists such as Deci and Ryan (1985, 2000). The theory is based on positive psychology and emphasizes the active role of the individual in the process of motivation and the role of the external environment in hindering or facilitating the formation of individual motivation (Ryan and Deci, 2000; Ntoumanis, 2005) and believes that motivation is a process of dynamic adjustment from nothing to something. Self-determination refers to the individual's free choice of behavior based on full awareness of personal needs and environmental information (Deci and Ryan, 1985). SDT is applied to the field of education and focuses on increasing students' autonomy and motivation, improving teaching effectiveness, and promoting the development and progress of individual students. Basic needs theory (BNT) is the core theory of SDT. It is believed that there are three basic psychological needs in individuals: autonomy, competence, and relatedness. These are inherent and innate in human beings and are environmental characteristics that promote individual motivation. When the external environment satisfies these three basic psychological needs, it can promote the excitation of intrinsic motivation and the internalization of external motivation, that is, to form a high degree of self-determination motivation and to learn more actively.

### 1.2. Flipped classroom

The enlightenment of self-determination theory for classroom teaching in local colleges and universities: try to create conditions for students to meet their three basic psychological needs of autonomy, competence, and relationship; promote students to generate more intrinsic motivation; or internalize external motivation, to improve the enthusiasm and initiative of students' individual learning. The need for autonomy refers to students' active participation in teaching and learning activities without being coerced by external factors and out of their own decision. Competence needs refer to students' perceptions of their ability to perform tasks in the learning process. Relational needs refer to the individual's perception of the emotional bonds established with significant others during the activity (Fan and Long, 2022). In the teaching and learning process, relational needs refers to individuals having harmonious and cordial relationships and sincere emotional interactions with teachers and classmates, and members helping each other, working in solidarity, and having a sense of belonging (e.g., support, trust, and care). With the advent of the “Internet+” era, the teaching mode is now showing a diversified trend. The flipped classroom teaching mode has changed the roles of teachers and students in traditional teaching by reversing the arrangement of knowledge transfer and knowledge internalization and re-planning the use of classroom time. It is a revolution of the traditional teaching mode. Its essence is that the student's complete knowledge learning independently before class and realize knowledge transfer, so as to give full play to students' subjective initiative; teachers organize students to communicate, discuss, and answer questions in class and provide space and time for teachers and students to interact, so as to achieve internalization of knowledge. Jonathan Bergmann and Aaron Sams found that the flipped classroom returns the main body of learning to the students, and the greatest benefit is the increase in teacher–student, student–teacher, and student–student interaction, which gives full play to the students' subjectivity (Bergmann and Sams, 2012). In the process of student–teacher interaction, students can feel the teacher's care, support, and understanding. At the same time, teachers can experience students' trust, respect, and love for them, and a strong emotional connection is established between teachers and students. Interaction promotes mutual communication between each other, develops a sense of cooperation and the ability to work together, and finds a sense of belonging in the collective. In addition, interactive, cooperative learning is also conducive to students to form a richer and more comprehensive understanding of knowledge, while training students' thinking ability. As can be seen, the flipped classroom takes meeting students' basic psychological needs as an implementation strategy for the entire teaching process, effectively stimulates students' autonomous motivation for learning and promotes the internalization of external motivation, which enhances classroom teaching efficiency. The effectiveness of the flipped classroom model for the teaching and learning process has been investigated by a significant body of research, across different subject domains and educational levels (Schultz et al., 2014; Lo and Hew, 2017; Sergis et al., 2018; Khayat et al., 2020).

### 1.3. Mobile application

**Figure 1:**
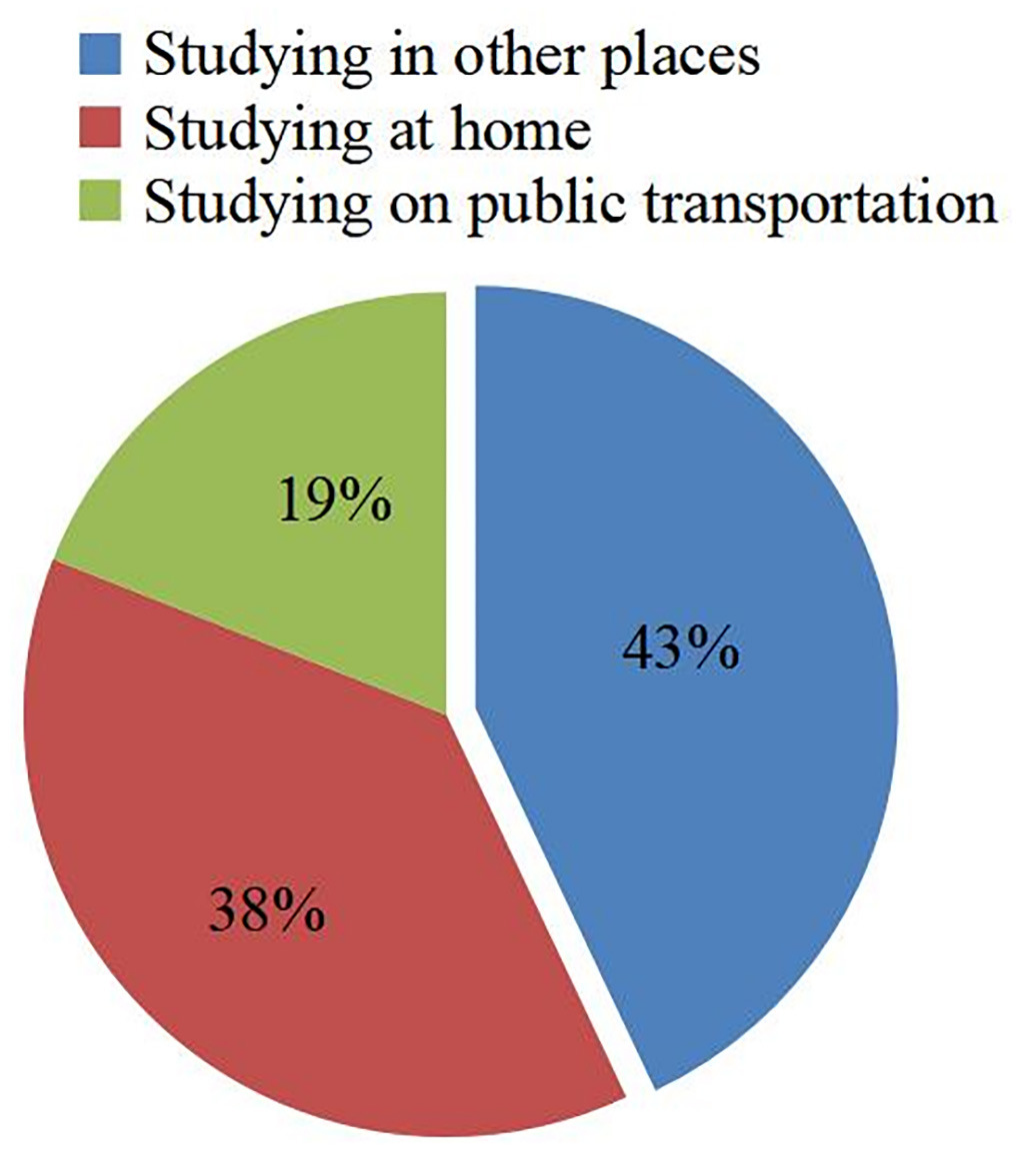
Non-classroom learning for mobile applications users.

In today's world, with the rapid development of science and technology, all kinds of smart devices flood people's lives. Being easily connected to the Internet, mobile devices such as laptops, smart phones, and tablets, which have small sizes and are really convenient to take are widely used by people. Especially, the popularization of smart phones makes the devices have become an indispensable part of our daily life. Until the year 2021, with the improvement of the functions of mobiles and the decrease in their price, there are over 70% of people all over the world use smart phones and 90% of the population use mobile broadband networks. The popularity of mobile devices has made various application software programs. Currently, mobile terminals, including mobile phones and tablets, enable mobile applications to be used in every field such as medical treatment, commerce, and education (Gayatri and Subrahmanyam, 2021; Khrais and Alghamdi, 2021; Kirsi et al., 2021; Seo et al., 2021; Wang and Qi, 2021). People can use applications with their mobile devices everywhere, which can bring tremendous changes in the informatization of society. In terms of mobile applications, educational applications have the most development prospects and market potential. College students have a strong receptive ability to new things, so the use of electronic devices for learning has become very common for them. According to a survey of 500 learners who have smart phones or tablets, it is found that 96% of them had non-classroom learning experiences, among them, 38% studied at home, 19% studied on public transportation, and 43% studied in other places (Figure 1). The results suggest that learners use mobile applications on a large scale to learn anytime and anywhere, and the applications make a fundamental change in their way to learn. When mobile application appears in classroom teaching, it expands the learning space and personalizes learning services for learner; when designing the courses for college students with mobile applications as teaching methods and means, it has not only changed the traditional learning method, which fundamentally changed the learning environment and conditions, but also changed the original passive state of learners and turned them into the master of learning behaviors (Liu et al., 2015). In the background of advancing the deep integration of information technology and education, mobile applications are regarded as a means of information technology. In addition, because smart terminals such as smart phones are easier to introduce into the teaching process, various mobile learning platforms have emerged. SDT emphasizes the interaction between the external environment and individual learning. If the two are harmonious and unified, it will play a promoting role, otherwise, it will form a block. These mobile learning platforms are the carriers of knowledge and emotional interaction between teachers and students and play an important intermediary role. The use of these platforms has great significance to promote the realization of learner-centered classroom teaching and is also conducive to the cultivation of innovation and high-quality applied talents in local colleges under the conditions of informationization. Therefore, the construction of a convenient, flexible, and efficient mobile learning platform can gradually promote students' motivation and internalization of learning. The autonomous mobile learning methods based on mobile terminals such as smart phones as the carrier are gradually being applied in the education field, which can create excellent conditions for everyone to realize lifelong learning, and will also become the main development direction of knowledge learning in the future (Brown, 2005; Walsh, 2015).

### 1.4. Purpose of this study

Research on the effective integration of mobile applications and flipped classroom is of great significance to promoting the development of educational informatization. It has changed the teaching and learning mode of traditional classroom and provided new development ideas for education and teaching reform. Therefore, combined with the structural mechanics course of civil engineering, this study specifically introduces the construction and implementation of the flipped classroom teaching mode based on mobile applications and motivated by the SDT perspective, the present study aimed to investigate the degree of satisfaction of students' basic psychological needs in the process of learning under the flipped classroom model based on mobile applications, as well as students' satisfaction with this classroom model. The three research questions proposed were as follows: (1) Can the flipped classroom model based on mobile applications meet the basic psychological needs of students during learning, that is, the needs of autonomy, competence, and relatedness? (2) Does the flipped classroom model based on mobile applications help improve students' classroom satisfaction in the process of learning? (3) What is the relationship between students' basic psychological needs and classroom satisfaction in this classroom model?

## 2. Construction of flipped classroom model based on mobile application

### 2.1. The mobile learning platform

Mobile applications will be applied to teaching process to form various learning platforms. The ways of constructing can be divided into two types: mobile learning platform based on WeChat and independently developing mobile application learning platform. Mobile learning platforms based on WeChat will be divided into three types: WeChat group, WeChat Official Accounts, and WeChat Mini programs (Hou et al., 2017). WeChat groups are built to add members to form a small user group, which can conduct real-time chat, language calls, video transmission, test, and picture link transmission to realize the sharing of learning materials (Wang, 2013), but the function of APP supporting cannot be established. Official Accounts of WeChat, registering by person or industry, can be designed as a mini mobile course platform (Lu, 2014). By using official accounts and subscriptions to push messages, automatic replies, and support videos, files, and link sharing, many functions such as setting personal information, real-time messages, users and materials management, group messages, and advanced setting can be realized (Liu, 2013), but it is limited in operation and interaction. Mini programs run on WeChat platform, which is a lightweight mobile application. It can realize the functions supported by an independent APP and have the advantages of no downloading, easy access, easy promotion, etc. The functions of independently developing APP learning platform are rich. It has swift response, but it is required to develop the application for IOS and Andriod users and the application needs to be updated from time to time. Duifene Teaching platform is a new teaching platform for Chinese teachers based on the WeChat official accounts. It has a simple interface, is easy to learn, and free to use. It is suitable for various teaching modes. The article uses this official account as the mobile application and platform of Flipped Classroom. In practical teaching, students can access the platform and join teaching activity by scanning the bar code with their mobile devices.

### 2.2. Design of teaching mode

Teaching mode is a relatively stable teaching activity structure framework and process established under the guidance of certain teaching ideas and teaching theories, and it reflects the simplified form of teaching activities (Wang, 2016). The main task of local colleges is to serve the regional economic and social development and to cultivate high-quality talents for the locality. Therefore, their teaching mode cannot divorce from this fundamental task. The guiding ideology of “teacher as the leading and student as the main body” will not change, and the goal of cultivating students' independent inquiry ability and innovation ability will not change. The combination of the benefits of traditional learning mode and Internet learning mode should be considered. The teaching mode not only should enable teachers to play a major role in guiding, enlightening, and monitoring process but also to fully reflect the initiative, enthusiasm, and creativity of students as the main body of learning. Based on mobile application, Flipped Classroom satisfies these concepts, which regards the students as center and deeply combines information technology and education.

**Figure 2:**
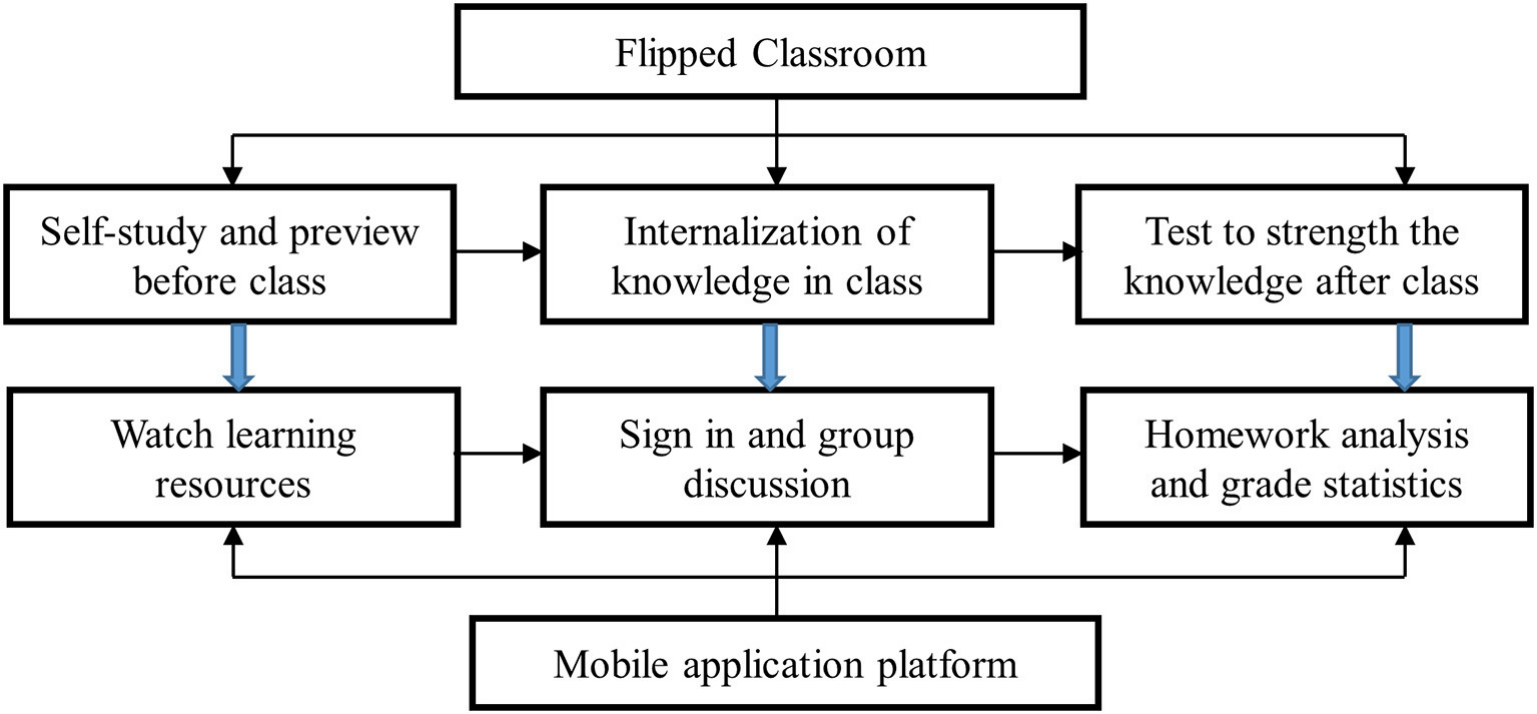
Design of the flipped classroom teaching mode based on mobile application.

According to the theory of Flipped Classroom, the teaching process is divided into pre-class, in-class and after-class: students learn the new knowledge and review before class and teachers publish the learning tasks, build and upload teaching resources; in class, students do group discussions, exchange results, and teachers' answer questions and provide personalized guidance and strengthen the internalization of knowledge; after class, students should do the assignment and test to strengthen the knowledge learned in class and teachers assign homework, analyze the homework and do score statistics, and improve learning materials. These activities will depend on the mobile learning platform. The design steps of flipped teaching mode based on WeChat application are shown in Figure 2. Mobile learning can realize real-time study and allow students to make full use of fragmented learning time to improve students' learning efficiency and effective learning time.

### 2.3. The realization of teaching mode

**Figure 3:**
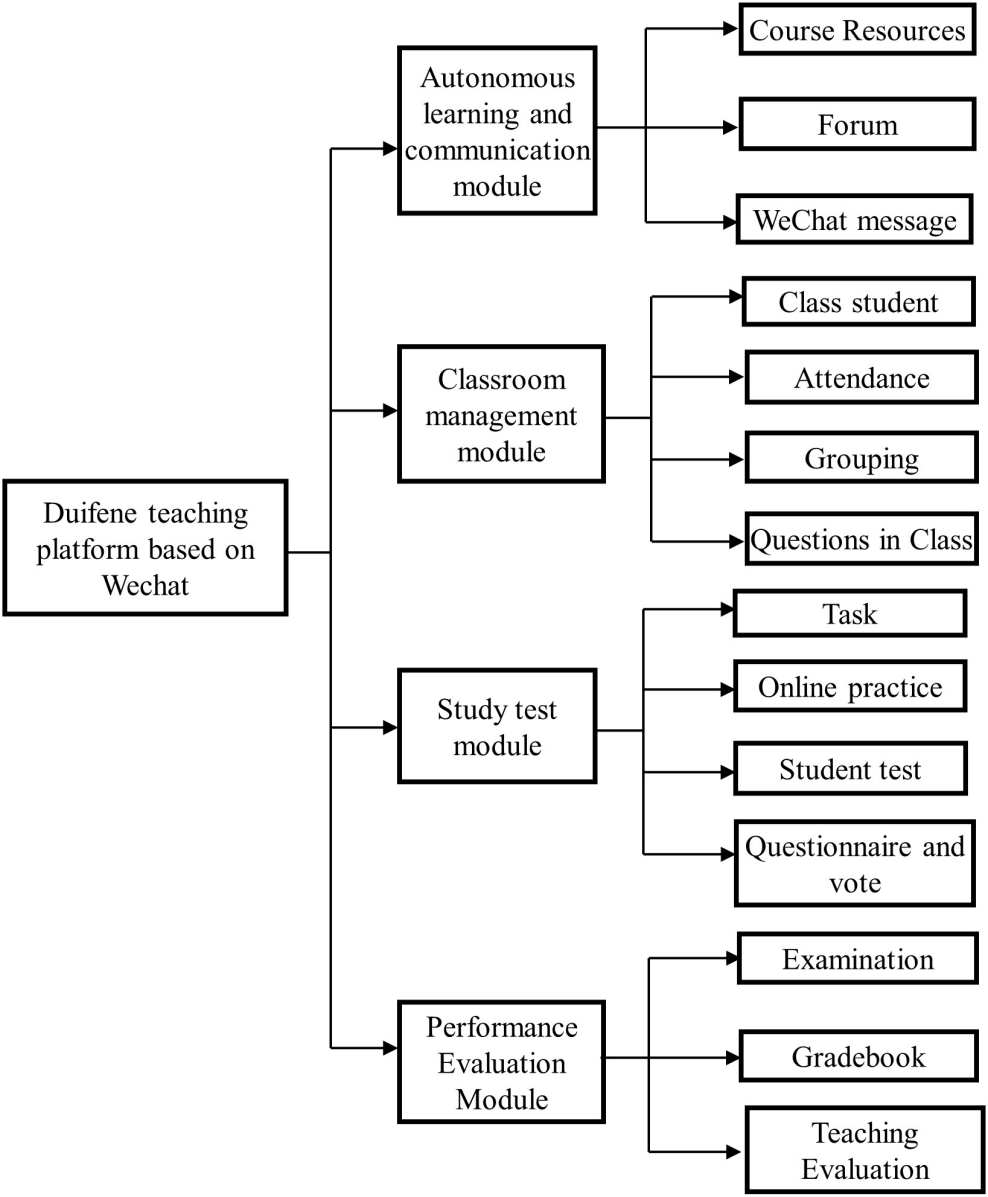
The function structure provided by the Duifene teaching platform.

The Duifene teaching platform based on WeChat official accounts provides teachers and students with many functions including class student management, homework, attendance statistics, grouping, course resources, online exercise, discussion areas, classroom questions, questionnaires, voting activities, transcripts, WeChat messages, and teaching evaluation. It supports teachers and students to simultaneously use all the functions and services of the Duifenyi platform on computers and mobile phones. The structure of teaching function provided by Duifenyi is shown in Figure 3, we can select corresponding function modules according to course content, knowledge structure, and teaching design of the Flipped Classroom.

## 3. Practice of flipped classroom model based on mobile application

The article takes the course of “Structural Mechanics” in civil engineering as the background to implement and use this teaching mode. First, teachers will upload the relevant materials (PPT, video, and teaching plan) of knowledge points in the classroom to the WeChat public platform before class, so that students can use the fragmented time after class to preview or watch new knowledge to meet the autonomous needs of students; then teachers will organize some activities such as group discussions and assignments in class to achieve internalization of knowledge for students in class; finally, teachers assign the homework and test after class to detect the mastery of students' knowledge and understand the teaching effect. The aforementioned teaching process uses the mobile application platform to closely focus on the three major psychological needs of individual students to enhance their learning effects.

Then, Chapter 5 “Three Hinged-Arch” in the course of “Structural Mechanics” will be taken as an example to briefly explain the application steps of the flipped classroom teaching mode.

### 3.1. Pre-class

**Figure 4:**
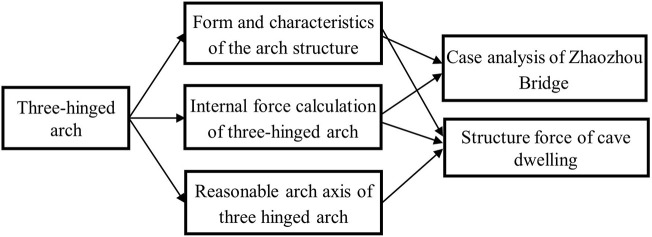
The relationship diagram between the three-hinged arch knowledge module, knowledge points and case projects.

When teachers prepare for this lesson, they will send the videos, PPT, and related teaching animations about “three hinged arch” part to the teaching resources of the Duifenyi learning platform. One week before the course, a learning announcement was issued through the WeChat public platform. Students use their fragmented time after class to preview or watch to complete the learning of pre-class knowledge. Video content is organized based on knowledge points, so that students can quickly view the required content through mobile intelligent terminal devices such as mobile phones when they are previewing, learning, and reviewing. The videos are short but useful and their durations are limited within 10 min to prevent distraction. In view of the characteristics of the structural mechanics course that emphasizes both theory and practice, the knowledge points taught in the pre-class video are integrated into actual engineering cases in order to do the in-class training, stimulate students' interest in learning, and cultivate students' inquiry ability. The organization relationship between the knowledge module, knowledge points, and case projects in the three-hinged arch course is shown in Figure 4.

### 3.2. In class

First of all, the class will be created by Duifene teaching platform and the system will establish a QR code. When students have class, the QR code will be shared with them. Students should scan it to enter the class group of structural mechanics. They will use the sign-in function to complete class attendance when they have class. According to the teaching mode of Flipped Class, students have mastered the basic knowledge of three-hinged arch. In class, the dividing group function will divide the class into eight studying group to discuss the mechanics knowledge and mechanical properties contained in the representative cases of the two arch structures “Zhaozhou Bridge” and “Cave Dwellings”. First, the members of each group will share their views based on their preview and form a unified point of view, which will be explained by the representatives of each group. During the explanation, other groups can make supplement and exchange their ideas to ensure that all students in the class have comprehensive and accurate knowledges. Practical engineering cases can help students master and understand what they have learned and improved their ability to integrate theory with practice. This is the entry point for cultivating high-quality applied talents. After the session, teacher should comment or correct the explanation to ensure the correctness of the knowledge points and promote their internalization and improvement of knowledge.

### 3.3. After class

After students do self-learning before class and internalization of in-class knowledge, they should consolidate the knowledge. Teachers can assign some homework to help students to consolidate the learning results of this section. Students upload the homework results with the help of the Duifene learning platform, and the teacher will give a summary evaluation according to the completion of the homework, so that students can check for omissions. Teachers can also allow students to complete the knowledge point test in this section on the platform and understand the problems existing in student based on their test results. They can analyze the test results and feedback them in the WeChat official account, and students can find their weak points of “three-hinged arch” by receiving feedback, so as to strengthen the learning of relevant knowledge points again.

## 4. Research methods

### 4.1. Research subjects

One hundred and fifty-one students from a local university in the northwest of China participated in this survey. All of these students have experienced the flipped classroom model based on mobile application, among which the number of freshmen, sophomores, juniors, and seniors are 1, 3, 112, and 35, respectively, 37 female and 114 male students. The number of students whose majors are in natural sciences is 131, and the number of students in humanities and social science and others is 20. Due to the small number of freshmen, they were screened out during the analysis.

### 4.2. Instrument

The questionnaire used in this study consisted of three parts, namely, background information about the subjects, basic psychological needs questionnaire, and classroom satisfaction questionnaire. The Activity-Feelings States (AFS scales), which was developed by Reeve and Sickenius (1994), was used to measure students' basic psychological needs satisfaction and was shown to have good reliability and validity (Reeve and Sickenius, 1994). The questionnaire contains four dimensions: autonomy (four items), competence (three items), relatedness (three items), and tension (three items), with a total of 13 items. Although tension is not a psychological need, it was considered to be relevant because tension serves as an emotional marker of internal motivations that are antagonistic to intrinsic motivation (Ryan et al., 1991). In laboratory studies, tension correlates negatively with intrinsic motivation but positively with anxiety-related internal motivations, such as ego-involvement, frustration, and the Zeigarnik effect (Ryan et al., 1991). Cronbach's α of internal consistency reliability of the scale in this study was 0.936. Students' satisfaction with the new classroom model was measured by using the classroom satisfaction indicator on the student learning engagement scale of “flipped classroom” model (Zhao et al., 2021), with a total of four items and internal consistency reliability of 0.958. All questionnaires in this study were scored on a seven-point Likert scale.

### 4.3. Data collection and analysis

The researcher first entered the aforementioned questionnaire into the Questionnaire Star platform, a WeChat QR code was generated, and then commissioned the teacher to share the QR code with students who had participated in the flipped classroom model based on mobile application. After obtaining students' consent, students were finally asked to complete the questionnaire during class. The researcher collected 151 questionnaires and finally collated 151 valid questionnaires. Then the researcher imported the questionnaires into SPSSAU for descriptive statistical analysis, variance analysis, correlation analysis, and regression analysis in order.

## 5. Findings and analysis

### 5.1. Basic information about the study variables

#### 5.1.1. The basic situation of students' basic psychological needs satisfaction in the flipped classroom model based on mobile application

Since the scale is scored on a seven-point Likert scale, there is a 1–7 response scale (strongly disagree to strongly agree) for each item. “Strongly disagree” is scored as “1 point”, “Disagree” is scored as “2 points”, “Somewhat disagree” is scored as “3 points”, “uncertain” is scored as “4 points”, “agree” is scored as “5 points”, “Somewhat agree” is scored as “6 points”, and “strongly agree” is scored as “7 points”. Thus, each measure involving basic psychological needs satisfaction has a theoretical neutral value of 4 points. Students' basic psychological needs satisfaction in this classroom model is shown in Supplementary Table 1.

From the descriptive statistics of students' basic psychological needs satisfaction shown in Supplementary Table 1, the absolute values of kurtosis and skewness of the whole sample data are < 3, indicating that the data are normally distributed. The average values of students' sense of autonomy, competence, relationship, and basic psychological needs satisfaction in this classroom teaching mode are significantly higher than the median score of 4, which indicates that the basic psychological needs satisfaction of the surveyed students is in the middle to upper level. The average score of 4.796 for the sense of tension indicates that students recognized that this classroom model would bring some stress and challenge to them. In terms of detection rate, 92.72% of students with a mean score of basic psychological needs satisfaction greater than or equal to 4, and 7.28% of students with a score below 4. The detection rates of the three dimensions of students' basic psychological needs satisfaction: autonomy, competence, and relatedness with a mean value greater than or equal to 4 were 89.4, 94.7, and 96.03%, respectively, and the average value of tension with a score > 4 reached 77.48%, while only 22.52% were below 4. Therefore, it can be assumed that the basic psychological needs of the surveyed students are satisfied to a high degree. However, this classroom model also makes them feel stressed and anxious.

#### 5.1.2. Students' satisfaction with this classroom model

From the descriptive statistics of students' classroom satisfaction shown in Supplementary Table 2, the absolute values of kurtosis and skewness are < 3, and the whole sample data are normal. The average values of students' satisfaction with the teacher's classroom teaching behavior as well as the classroom model are significantly higher than the median value 4, indicating that the satisfaction situation of the surveyed students with the flipped classroom model based on mobile application is in the middle to upper level. In terms of the detection rate, 90.07% of the students had an average satisfaction score greater than or equal to 4, and only 9.93% of the students had a mean score lower than 4. Therefore, it can be concluded that the surveyed students are satisfied with this classroom model to a high degree.

### 5.2. Differences in study variables in terms of demographic characteristics

#### 5.2.1. Satisfaction of students' basic psychological needs

Analysis of variance was used to study the differences in students' basic psychological needs satisfaction under the flipped classroom model based on mobile application in terms of the students' gender, grade, major, and interest of major. As seen in Supplementary Table 3, there was no significant difference in students' basic psychological needs satisfaction by gender, grade, and major, with *p*-values greater than 0.05. The difference in students' basic psychological needs satisfaction on interest of major was relatively significant, here F = 4.391, *p* = 0.005, reaching 0.01 level of significance.

#### 5.2.2. Students' classroom satisfaction

The analysis of variance was used to examine students' classroom satisfaction with this model in terms of gender, grade, major, and degree of interest in their majors. As can be seen in Supplementary Table 4, the *p*-values were all greater than 0.05, indicating that there were no differences in students' classroom satisfaction in terms of gender, grade, major, and degree of interest in the major, all of which showed consistency.

### 5.3. Analysis of the relationship between students' classroom satisfaction and basic psychological needs satisfactions

#### 5.3.1. Correlation analysis of students' classroom satisfaction and basic psychological needs satisfaction

Correlation analysis was used to examine the correlations between classroom satisfaction and a total of four items: autonomy, competence, relatedness, and basic psychological needs satisfaction, respectively. The Pearson correlation coefficient was used to indicate the strength of the correlations. As shown in Supplementary Table 5, the correlation coefficient between students' classroom satisfaction and basic psychological needs satisfaction was 0.844 and showed significance at the 0.01 level. The correlation coefficients between students' classroom satisfaction and each sub-dimension such as autonomy, competence, and relatedness were 0.736, 0.809, and 0.805, respectively, and all of them reached the significance level of 0.01, which also showed a significant positive correlation. All of these coefficients are greater than 0.7, thus it can be concluded that there is a strong positive correlation between students' classroom satisfaction and basic psychological needs satisfaction.

#### 5.3.2. Regression analysis of students' classroom satisfaction and basic psychological needs satisfaction

The basic psychological needs satisfaction variable was generated by averaging the three dimensions of autonomy, competence, and relatedness by the “Generate Variable” function in the data processing of the software, and it has a strong autocorrelation with the three sub-dimensions themselves. Therefore, it could not be used as an independent variable in the regression analysis. Instead, its sub-dimensions of autonomy, competence, and relatedness were used as independent variables, and classroom satisfaction was used as the dependent variable in a linear regression analysis.

As seen in Supplementary Table 6, the regression coefficient values of autonomy, competence, and relatedness are 0.123 (*t* = 1.985, *p* < 0.05), 0.351 (*t* = 4.523, *p* < 0.01), and 0.409 (*t* = 5.966, *p* < 0.01), respectively, indicating that autonomy, competence, and relatedness all have a significant positive effect on classroom satisfaction. In contrast, relatedness (β = 0.413, *p* < 0.01) and competence (β = 0.370, *p* < 0.01) had a greater effect on classroom satisfaction. The coefficients of the model (R^2^ = 0.738, *F* = 138.101, *p* < 0.05) show that the model fits well. In addition, testing for multiple co-integrations of the model revealed that all the VIF values in the model were less than 5, implying that there was no co-integration problem, and the D-W values are around the number 2, thus indicating that the model does not have autocorrelation and the model is good.

## 6. Discussion

### 6.1. Characteristics of students' basic psychological needs satisfaction and classroom satisfaction under the flipped classroom model based on mobile applications

This study showed that the current tested students had a high level of basic psychological needs satisfaction under the flipped classroom model based on mobile applications, but there was also a sense of stress for the majority of the tested students. There were no significant differences in basic psychological needs satisfaction by gender, grade level, and major. It shows that the “student-centered” learning environment built by this mobile application-based flipped classroom model enables students to satisfy their basic psychological needs in the learning process to a certain extent. This psychological motivation leads to healthy psychology and positive learning behaviors (Mai and Liu, 2021). As stated in previous research results, if the external environment satisfies the three intrinsic needs of autonomy, competence, and relatedness, it will facilitate the internalization of external values and self-integration, which will be conducive to self-motivation and intrinsic motivation (Li, 2014). Therefore, the satisfaction of one's basic psychological needs is considered to predict positive behavior (Baard et al., 2004). At the same time, this classroom model has wide applicability, not limited by gender, major and students' grade level, and it is a new teaching model that can be widely popularized. However, there was a significant difference in the interest of major. That is to say, if students have a high interest in their majors, their learning motivation and autonomy will be high, and they will be more motivated in the learning process and have a higher sense of satisfaction with basic psychological needs; if students have no interest in their majors, their learning initiative will not be high and their sense of satisfaction with basic psychological needs will be low. Thus, the degree of psychological basic needs satisfaction differed significantly in the interest of major, which is consistent with the finding of a positive correlation between basic psychological needs, self-determination motivation, and initiative engagement (Fan and Long, 2022) and also in line with the research results of previous scholars (Joe et al., 2017; Alamer, 2021). Some of the subjects felt stressed because they were used to the traditional classroom where the teacher mainly taught and the students passively accepted, they were afraid of the teacher's authority, they had difficulties in teacher–student interaction and communication, and they spent more time learning new knowledge before class than in the traditional classroom.

The tested students were also highly satisfied with this classroom model. Classroom satisfaction does not show significant differences depending on gender, grade level, major, and degree of interest in the major. In other words, the tested students' classroom satisfaction with this model is consistent regardless of gender, grade level, majors, and whether they are interested in their major or not, with no significant differences. Therefore, the mobile application–based flipped classroom model is a popular and advantageous teaching model that can span different subject areas (Bergmann and Sams, 2012; Schultz et al., 2014; Lo and Hew, 2017; Sergis et al., 2018; Khayat et al., 2020).

### 6.2. Characteristics of the relationship between students' basic psychological needs satisfaction and classroom satisfaction

The results of the correlation analysis in this study showed that there was a high positive correlation between the satisfaction of basic psychological needs and classroom satisfaction among the students tested in the current study, and there was also a high positive correlation between each sub-dimension and classroom satisfaction, with correlation coefficients above 0.7. Therefore, the results of this study indicate that the higher satisfaction of students' basic psychological needs in this classroom model, the higher classroom satisfaction. Among them, autonomy, competence, and relatedness in the students' basic psychological needs satisfaction had significant effects on the regression of classroom satisfaction. In particular, competence and relatedness had a greater impact. It implies that students' sense of autonomy has a lesser impact on classroom satisfaction with this model. Scholars argue that environmental factors and individual differences affect autonomy (Gagne and Deci, 2005). Students can effectively integrate external rules if their external environment allows them to make free choices according to their wishes. Some students feel that this model has a large pre-class load, coupled with poor personal self-discipline, so they cannot complete their learning tasks autonomously. Therefore, they prefer to accept the traditional classroom. In addition, the learning resources on mobile application platforms are more fragmented and scattered, which are not as complete as the knowledge system taught in the traditional classroom. Chen et al. believes that fragmented learning is not conducive to students' in-depth learning, which reduces their learning autonomy (Chen et al., 2016). Finally, due to the short classroom time, the time available for each individual to communicate is limited, the interaction is not deep enough, and the personal views and ideas are not fully expressed, which reduces the students' initiative and motivation to participate in classroom activities. Thus, all of these lead to a slight decrease in the impact of autonomy on classroom satisfaction. When a student completes his learning task successfully and is satisfied with his performance in the learning process such as homework, group discussion, and teacher's questions and also receives praise from his classmates and teacher, then he will be filled with a sense of achievement inside and will be more satisfied with this classroom model. In addition, this classroom model increases teacher–student and student–student interaction, gives full play to students' subjectivity, allows students to communicate more in the learning process, establishes a more harmonious relationship with teachers and classmates, and finds a sense of belonging in the collective. As a result, students' classroom satisfaction is somewhat higher.

## 7. Conclusion and recommendations

With the rapid development of network information technology and the popularity of mobile Internet devices, the deep integration of information technology and education teaching process will become an inevitable trend. Mobile applications have already started to penetrate into the field of education and teaching. The flipped classroom model, which originated in the field of basic education in the United States, has attracted much attention. Combining mobile application with a flipped classroom, this study proposes a flipped classroom model based on mobile application to construct a student-centered supportive external learning environment to realize more transformation of internal motivation, which stimulates students' learning motivation and promotes internalization for individual development and progress. In this study, first, taking the course of Structural Mechanics in Civil Engineering as an example, the teaching implementation process of the flipped classroom based on WeChat's Counterpoint teaching platform is introduced in detail, and this mobile application–based flipped classroom model is practiced. Second, from the perspective of self-determination theory, students' basic psychological needs satisfaction under the mobile application–based flipped classroom model and students' satisfaction with this classroom model are analyzed, and the relationship between students' basic psychological needs satisfaction and classroom satisfaction is discussed. Finally, after statistical analysis, the results showed that this flipped classroom model based on mobile applications met students' psychological needs in the areas of autonomy, competence, and relatedness, and students' satisfaction with this classroom model was relatively high; the satisfaction of students' basic psychological needs and each sub-dimension in this model were highly positively correlated with classroom satisfaction, and the regression relationship between the sense of autonomy, competence, and relatedness and classroom satisfaction was significant. In particular, the regression effect of competence and relatedness on classroom satisfaction was greater. Therefore, the most direct way to increase classroom satisfaction is to enhance students' basic psychological satisfaction in classroom learning. As students with high basic psychological needs satisfaction can stimulate higher autonomous motivation and promote more active engagement in the learning process (Fan and Long, 2022). This finding is consistent with existing research results (Noels et al., 1999, 2019; Carreira, 2012; Alamer and Lee, 2019), that is, learners with high satisfaction of basic psychological needs exhibit higher autonomous motivation. Therefore, teachers should start by understanding the students' basic psychological needs and actively explore ways to enhance the satisfaction of students' psychological needs in order to improve their satisfaction with the classroom model. In this regard, the “teacher autonomy support” intervention project carried out by Cheon et al. is of reference significance (Cheon et al., 2018). In this project, teachers learn how to reduce control over students' behaviors and provide them with more independent support in the teaching process, which greatly improves the satisfaction of students' psychological needs. There are also external factors that cause improvements in the learning environment, such as learning materials, task design, student–teacher interaction, and classroom structure (Dincer et al., 2019; Mai and Liu, 2021; Sun and Wang, 2022), and these external factors may bring important effects on students' psychological needs satisfaction and self-determined motivation. However, in the process of implementing the flipped classroom model based on applications, there are still some problems that need to be considered and solved, such as how to motivate and initiate students with poor self-discipline; how to improve each student's participation, communication and expression ability, and teamwork spirit while enriching professional knowledge within the limited classroom time, and how to improve the mobile application function so that it can be better integrated with the flipped classroom teaching model. All these need to be further improved in the subsequent research and application work.

Due to the limitations of the sample and research conditions, there may be certain limitations in the findings of this study. In future research, the extension and connotation of students' basic psychological needs and classroom satisfaction should be further clarified, and the relationship between basic psychological needs and classroom satisfaction should be explored in depth in a larger sample size. In addition, more intuitive research materials should be obtained through classroom observation method and in-depth interview method to provide scientific reference for the development and innovation of classroom teaching mode.

## Data availability statement

The original contributions presented in the study are included in the article/Supplementary material, further inquiries can be directed to the corresponding author.

## Ethics statement

Ethical review and approval was not required for the study on human participants in accordance with the local legislation and institutional requirements. Written informed consent from the [patients/participants OR patients/participants legal guardian/next of kin] was not required to participate in this study in accordance with the national legislation and the institutional requirements.

## Author contributions

YH and YL: initial draft and methods. YL: revision and supervision. YH: analysis and interpretation. All authors contributed to the article and approved the submitted version.
