# Supplementary material for: Research and practice of flipped classroom based on mobile applications in local universities from the perspective of self-determination theory
Source: Front Psychol. 2023 Jan 9;13:963226. doi: 10.3389/fpsyg.2022.963226 (PMC9868744; doi:10.3389/fpsyg.2022.963226)
Supplement: Supplementary file 1 [file Table_1.docx]

Supplementary Material

| **Table 1** Basic situation of students’ basic psychological needs under the flipped classroom model based on mobile applications | | | | | |
| --- | --- | --- | --- | --- | --- |
| Variable | Average score | Standard deviation | Median score | Kurto | Skewness |
| Autonomy | 5.214 | 1.403 | 5.500 | 1.278 | -1.092 |
| Competence | 5.349 | 1.257 | 5.667 | 1.537 | -1.063 |
| Relatedness | 5.468 | 1.205 | 6.000 | 1.794 | -1.094 |
| Tension | 4.796 | 1.280 | 4.750 | -0.169 | -0.171 |
| Satisfaction of basic psychological needs | 5.330 | 1.196 | 5.600 | 1.772 | -1.040 |
