# Supplementary material for: Research and practice of flipped classroom based on mobile applications in local universities from the perspective of self-determination theory
Source: Front Psychol. 2023 Jan 9;13:963226. doi: 10.3389/fpsyg.2022.963226 (PMC9868744; doi:10.3389/fpsyg.2022.963226)
Supplement: Supplementary file 2 [file Table_2.docx]

Supplementary Material

| **Table 2** Basic situation of students' classroom satisfaction under the flipped classroom model based on mobile applications | | | | | |
| --- | --- | --- | --- | --- | --- |
| Variable | Average score | Standard deviation | Median score | Kurto | Skewness |
| Classroom satisfaction | 5.179 | 1.193 | 5.283 | 1.092 | -0.779 |
