# Supplementary material for: Research and practice of flipped classroom based on mobile applications in local universities from the perspective of self-determination theory
Source: Front Psychol. 2023 Jan 9;13:963226. doi: 10.3389/fpsyg.2022.963226 (PMC9868744; doi:10.3389/fpsyg.2022.963226)
Supplement: Supplementary file 3 [file Table_3.docx]

Supplementary Material

| **Table 3** Differences of students’ basic psychological needs in terms of demographic characteristics | | | | | | |
| --- | --- | --- | --- | --- | --- | --- |
| Analysis item | Demographic variables | Sample size | Average value | Standard Deviation | F-test | *p* |
| Gender | Male | 114 | 5.31 | 1.27 | 0.165 | 0.685 |
|  | Female | 37 | 5.40 | 0.93 |  |  |
| Grade | Sophomore | 3 | 5.80 | 0.20 | 1.113 | 0.331 |
|  | Junior | 112 | 5.39 | 1.18 |  |  |
|  | Senior | 35 | 5.08 | 1.29 |  |  |
| Majors | Natural Sciences | 131 | 5.37 | 1.23 | 0.599 | 0.551 |
|  | Humanities and Social Sciences | 13 | 5.19 | 0.98 |  |  |
|  | Other | 7 | 4.90 | 0.97 |  |  |
| Interest of Major | Very interested | 11 | 6.27 | 0.87 | 4.391 | 0.005** |
|  | Interested | 79 | 5.45 | 1.15 |  |  |
|  | commonly | 55 | 4.99 | 1.18 |  |  |
|  | uninterested | 6 | 5.17 | 1.55 |  |  |
| Note: * represents p value＜0.05, ** represents p value＜0.01 | | | | | | |
