# Supplementary material for: Research and practice of flipped classroom based on mobile applications in local universities from the perspective of self-determination theory
Source: Front Psychol. 2023 Jan 9;13:963226. doi: 10.3389/fpsyg.2022.963226 (PMC9868744; doi:10.3389/fpsyg.2022.963226)
Supplement: Supplementary file 4 [file Table_4.docx]

Material Supplementary

| **Table 4** Differences of students’ classroom satisfaction in terms of demographic characteristics | | | | | | |
| --- | --- | --- | --- | --- | --- | --- |
| Analysis item | Demographic variables | Sample size | Average value | Standard deviation | F-test | *p* |
| Gender | Male | 114 | 5.20 | 1.27 | 0.111 | 0.739 |
|  | Female | 37 | 5.12 | 0.94 |  |  |
| Grade | Sophomore | 3 | 5.80 | 0.23 | 0.664 | 0.516 |
|  | Junior | 112 | 5.20 | 1.18 |  |  |
|  | Senior | 35 | 5.04 | 1.29 |  |  |
| Majors | Natural Sciences | 130 | 5.21 | 1.24 | 0.529 | 0.591 |
|  | Humanities and Social Sciences | 13 | 5.00 | 0.81 |  |  |
|  | Other | 7 | 4.81 | 0.91 |  |  |
| Interest of Major | Very interested | 11 | 5.75 | 1.21 | 1.355 | 0.259 |
|  | Interested | 78 | 5.22 | 1.16 |  |  |
|  | commonly | 55 | 4.99 | 1.21 |  |  |
|  | uninterested | 6 | 5.29 | 1.47 |  |  |
| * *p*<0.05 ** *p*<0.01 | | | | | | |
