# Supplementary material for: Research and practice of flipped classroom based on mobile applications in local universities from the perspective of self-determination theory
Source: Front Psychol. 2023 Jan 9;13:963226. doi: 10.3389/fpsyg.2022.963226 (PMC9868744; doi:10.3389/fpsyg.2022.963226)
Supplement: Supplementary file 5 [file Table_5.docx]

Supplementary Material

| **Table 5** Correlation coefficient matrix between students' basic psychological needs and classroom satisfaction | | | | | |
| --- | --- | --- | --- | --- | --- |
|  | Classroom Satisfaction | Relatedness | Competence | Autonomy | Satisfaction of basic psychological needs |
| Classroom Satisfaction | 1 |  |  |  |  |
| Relatedness | 0.805** | 1 |  |  |  |
| Competence | 0.809** | 0.780** | 1 |  |  |
| Autonomy | 0.736** | 0.711** | 0.804** | 1 |  |
| Satisfaction of basic psychological needs | 0.844** | 0.882** | 0.929** | 0.938** | 1 |
| * *p*<0.05 ** *p*<0.01 | | | | | |
