# Supplementary material for: Research and practice of flipped classroom based on mobile applications in local universities from the perspective of self-determination theory
Source: Front Psychol. 2023 Jan 9;13:963226. doi: 10.3389/fpsyg.2022.963226 (PMC9868744; doi:10.3389/fpsyg.2022.963226)
Supplement: Supplementary file 6 [file Table_6.docx]

Supplementary Material

| **Table 6** Linear regression between students' basic psychological needs and classroom satisfaction | | | | | | | | | |
| --- | --- | --- | --- | --- | --- | --- | --- | --- | --- |
|  | Unstandardized Coefficients | | Standardized Coefficients | t | p | VIF(variance inflation factor) | R^2^ | Adjust R^2^ | F-test |
|  | B | standard error | β |  |  |  |  |  |  |
| Constant | 0.425 | 0.241 | - | 1.762 | 0.080 | - | 0.738 | 0.733 | 138.101,p=0.000 |
| Autonomy | 0.123 | 0.062 | 0.145 | 1.985 | 0.049* | 2.984 |  |  |  |
| Competence | 0.351 | 0.078 | 0.370 | 4.523 | 0.000** | 3.761 |  |  |  |
| Relatedness | 0.409 | 0.069 | 0.413 | 5.966 | 0.000** | 2.689 |  |  |  |
| Dependent variable: classroom satisfaction | | | | | | | | | |
| D-W(Durbin-Watson): 1.810 | | | | | | | | | |
| * p<0.05 ** p<0.01 | | | | | | | | | |
